# Autoantibody detection is not recommended for chronic pancreatitis: a cross-sectional Study of 557 patients

**DOI:** 10.1186/s12876-019-0947-7

**Published:** 2019-02-14

**Authors:** Xiang-Peng Zeng, Ting-Ting Liu, Lu Hao, Lei Xin, Teng Wang, Lin He, Jun Pan, Dan Wang, Ya-Wei Bi, Jun-Tao Ji, Zhuan Liao, Ting-Ting Du, Jin-Huan Lin, Di Zhang, Hong-Lei Guo, Hui Chen, Wen-Bin Zou, Bai-Rong Li, Zhi-Jie Cong, Li-Sheng Wang, Zheng-Lei Xu, Ting Xie, Ming-Hao Liu, An-Mei Deng, Zhao-Shen Li, Liang-Hao Hu

**Affiliations:** 1 Department of Gastroenterology, Gongli Hospital, The Second Military Medical University, Shanghai, China; 2 Department of Gastroenterology, Changhai Hospital, The Second Military Medical University, Shanghai, China; 3 Department of Laboratory Medicine, Changhai Hospital, The Second Military Medical University, Shanghai, China; 4 Department of Gastroenterology, Hainan Branch of Chinese PLA General Hospital, Sanya, China; 5 Digestive Endoscopy Center, Changhai Hospital, The Second Military Medical University, Shanghai, China; 6 Department of Gastroenterology, Air Force General Hospital, Beijing, China; 7 Department of General Surgery, Renji Hospital, Shanghai Jiaotong University, Shanghai, China; 8 Department of Gastroenterology, Shenzhen People’s Hospital, Shenzhen, China; 9 Department of Gastroenterology, Zhongda Hospital, Southeast University, Nanjing, China; 10 Department of Gastroenterology, PLA Rocket Force Characteristic Medical Center, Beijing, China

**Keywords:** Chronic pancreatitis, Autoantibody, Autoimmune pancreatitis, Anti-β2-glycoprotein I antibody, Diabetes mellitus

## Abstract

**Background:**

Autoimmune factor was regarded as one of the risk factors in the pathogenesis of chronic pancreatitis (CP), especially for autoimmune pancreatitis (AIP). However, whether autoimmune factor plays a role in non-AIP CP or not was unknown.

**Methods:**

Hospitalized patients with non-AIP CP from January 2010 to October 2016 were detected for 22 autoantibodies at the time of hospital admission. Autoantibodies with frequency > 0.5% were enrolled to calculate the frequency in historial healthy controls through literature search in PubMed. Differentially expressed autoantibodies were determined between patients and historial healthy controls, and related factors were identified by multivariate logistic regression analysis.

**Results:**

In a total of 557 patients, 113 cases were detected with 19 kinds of positive autoantibodies, among them anti-β2-glycoprotein I (β2-GPI) antibody was most frequent (9.16%). Compared with historial healthy controls, the frequencies of serum β2-GPI and anti SS-B antibody in patients were significantly higher, while frequencies of anti-smooth muscle antibody and anticardiolipin antibody were significantly lower (all *P* < 0.05). Multivariate logistic regression analysis result showed that diabetes mellitus (OR = 2.515) and common bile duct stricture (OR = 2.844) were the risk factors of positive β2-GPI antibody in patients while diabetes mellitus in first−/second−/third-degree relatives (OR = 0.266) was the protective factor. There were no related factors for other three differentially expressed autoantibodies.

**Conclusions:**

Four autoantibodies were expressed differentially between patients with non-AIP CP and historial healthy controls. Due to limited significance for diagnosis and treatment of chronic pancreatitis, autoantibodies detection is not recommended conventionally unless suspected of AIP.

**Electronic supplementary material:**

The online version of this article (10.1186/s12876-019-0947-7) contains supplementary material, which is available to authorized users.

## Background

Chronic pancreatitis (CP) is a progressive inflammatory disease with irreversible destruction of the pancreatic parenchyma and ductal structures [1–5]. Autoimmune factor was regarded as one of the risk factors in the pathogenesis of CP. In 1995, Yoshida et al. [6] proposed the concept of autoimmune pancreatitis (AIP) to define this particular type of CP. Now autoimmune is regarded as the major definite pathogenesis of AIP, but whether autoimmune factor plays a role in non-AIP CP or not was unknown. Presence of autoantibodies has been included in Japan [7], Korean [8] and Asian [9] clinical diagnostic criteria for AIP, but clinical practice guidelines [10, 11] for CP had not clearly recommended whether autoantibody detection should be a conventional laboratory test or not. In the past few years, novel specific antibodies have emerged to help the diagnosis and differential diagnosis of AIP [12–14]. However, there have been no researches for specific antibodies of non-AIP CP. In the present study, we prospectively detected 22 common autoantibodies in 557 non-AIP CP patients to initially investigate the clinical significance of autoantibody detection.

## Methods

### Patient selection

Patients with CP admitted to the Department of Gastroenterology, Changhai Hospital for the first time from January 2010 to October 2016 were enrolled for the study mainly due to recurrent abdominal pain. For CP patients who did not experience pain, interventions were performed only when complications such as biliary stricture, pancreatic portal hypertension etc. had occurred, whereas diabetes mellitus and/or steatorrhea was not an indication for invasive treatment of CP. All patients underwent serum autoantibody detection at the time of hospital admission. Exclusion criteria were that: 1. Patients were formerly definitely diagnosed with AIP. 2. Combined with systemic lupus erythematosus, rheumatoid arthritis (RA), leukoderma, uarthritis or other autoimmune diseases before admission. 3. Autoimmune diseases were newly diagnosed within thirty days after autoantibody detection.

### Data collection and detection of autoantibodies

The following information was were prospectively collected: demographic data (age, sex, birthplace, et al), course of CP, medical history, history of other diseases, smoking and alcohol history, family history of pancreatic diseases and diabetes mellitus, laboratory and imaging findings, and treatment strategy.

Peripheral venous blood samples were obtained from all enrolled patients at admission to detect 22 common autoantibodies (EUROIMMUN Medical Laboratory Diagnostics Stock Company, Lübeck, German), whose sensitivity and specificity were provided in (see Additional file 1: Table S1). The distribution tested prior to using parametric tests of all autoantibodies was normal. Serum anti-double-stranded DNA (anti-ds DNA) antibody, anti-single-stranded DNA (anti-ss DNA) antibody, anti-SM antibody, anti-ribonucleoprotein (anti-RNP) antibody, anti SS-A antibody, anti SS-B antibody, anti-Jo-1 antibody, anti-Scl 70 antibody, anti-proliferating cell nuclear antigen antibody, anti-nucleosome antibodies, anti-histone antibody (AHA), anti-ribosomal antibody and anti-PM-Scl antibody were measured by EUROLINE according to the manufacturers’ instructions. Serum samples were tested for anti-smooth muscle antibody (SMA), anti-mitochondrial antibody (AMA), anti-neutrophil cytoplasmic antibody and anti-neutrophil perinuclear antibody by indirect immunofluorescence. Serum anti-glomerular basement membrane antibody, anti-proteinase 3 IgG antibody, anti-myeloperoxidase antibody, anti-β2-glycoprotein I (β2-GPI) antibody and anticardiolipin (ACL) antibody were measured by enzyme-linked immunosorbent assay.

### Search strategy

In order to compare the frequency of autoantibodies between non-AIP CP patients and healthy controls, a literature search for autoantibodies with frequency > 0.5% was performed through PubMed to identify eligible studies published. These literatures had to report the total positive rate of target autoantibody in historial healthy controls, and those literatures which only report the positive rate of some isoforms were excluded. These search strategy, which were defined prospectively, are provided in (see Additional file 1: Table S2). Then the frequency of target autoantibody in historial healthy controls was calculated by sum of cases in all enrolled studies.

### Definitions

The diagnosis of CP was established according to the Asia-Pacific consensus [15]. The diagnosis of AIP was established according to the Asian diagnostic criteria [9]. Alcoholic chronic pancreatitis (ACP) was diagnosed when alcohol intake exceeded 80 g/d for male and 60 g/d for female for at least two years in the absence of other causes, respectively [16, 17]. Heredity chronic pancreatitis was diagnosed when the CP patient had no less than two first-degree relatives with CP or recurrent acute pancreatitis, or no less than three second-degree relatives with CP or recurrent acute pancreatitis [18]. We defined abnormal anatomy of pancreatic duct system (including pancreas divisum and anomalous pancreaticobiliary junction) as an etiology of CP in our study, although it still remains a controversy [19]. A patient was defined as post-traumatic CP due to a definite history of abdominal trauma with imaging evidence of pancreatic injury and subsequent ductal dilation [20]. CP patients were considered idiopathic chronic pancreatitis (ICP) when none of the above etiologies were found.

### Statistical analysis

All analyses were performed using SPSS software (version 22.0, SPSS Inc.). Categorical variables were expressed as counts (percentages) and compared using the χ^2^ test, Fisher exact test or Mann-Whitney U test. Continuous variables were presented as mean ± standard deviation (SD). Multivariate logistic regression analysis was performed to identify the independent related factors of differentially expressed autoantibodies on the results of univariate analysis screening (factors with a significance level of *P* < 0.15 were included in the multivariate analysis). Odds ratio (OR) and 95% confidence interval (CI) were calculated. Statistical analyses were conducted at a significance level of 0.05 for all analyses.

## Results

### General characteristics of Study subjects

After exclusion of 98 patients, which consists of 91 patients diagnosed with AIP, 3 patients diagnosed with uarthritis, 1 patient diagnosed with leukoderma, and 1 patient newly diagnosed with RA, a total of 575 non-AIP CP patients were finally enrolled in the study, including 393 males and 164 females. Their general characteristics were presented in Table 1. The mean ± SD age at the onset and diagnosis of CP were 36.60 ± 16.59 and 41.53 ± 15.22 years old respectively. ICP was most common (70.4%) in this study.

**Table 1:** Clinical characteristics of 557 non-AIP CP patients

Items	Male (*n* = 393) n(%)	Female (*n* = 164) n(%)	Overall (*n* = 557) n(%)
Body mass index, kg/m^2a^	26.56 ± 105.94	20.89 ± 3.58	24.89 ± 89.01
Age at the onset of CP, y^a^	38.89 ± 15.86	31.09 ± 17.05	36.60 ± 16.59
Age at the diagnosis of CP, y^a^	43.60 ± 14.40	36.57 ± 16.00	41.53 ± 15.22
Etiology			
ACP	97(24.7)	15(9.1)	112(20.1)
ICP	266(67.7)	126(76.8)	392(70.4)
Abnormal anatomy of pancreatic duct	18(4.6)	11(6.7)	29(5.2)
Hereditary CP	11(2.8)	11(6.7)	22(3.9)
Post-traumatic CP	1(0.3)	1(0.6)	2(0.4)
Pancreatic stones	363(92.4)	153(93.3)	517(92.6)
Adolescent	40(10.2)	32(19.5)	72(12.9)
DM	102(26.0)	33(20.1)	135(24.2)
Steatorrhea	91(23.2)	41(25.0)	132(23.7)
Common bile duct stricture	40(10.2)	7(14.9)	47(8.4)
PPC	74(18.8)	20(12.2)	94(16.9)
SAP	13(3.3)	4(2.4)	17(3.0)
Onset manifestations			
Abdominal pain	320(81.4)	116(70.7)	436(78.3)
Pancreatic insufficiency	53(13.5)	35(21.3)	88(15.8)
Others	20(5.1)	13(7.9)	33(5.9)
Type of abdominal pain			
None	31(7.9)	26(15.9)	57(10.2)
Repeat attacks of acute pancreatitis	120(30.5)	45(27.4)	165(29.6)
Repeat pain	138(35.1)	57(34.8)	195(35.0)
Repeat acute attacks and pain	71(18.1)	25(15.2)	96(17.2)
Chronic pain	33(8.4)	11(6.7)	44(7.9)
Drinking history (g/d)			
0	223(56.7)	130(79.3)	353(63.4)
< 20	13(3.3)	5(3.0)	18(3.2)
20~80	53(13.5)	10(6.1)	63(11.3)
> 80	104(26.5)	19(11.6)	123(22.1)^b^
Smoking history, pack-year			
0	212(53.9)	130(79.3)	342(61.4)
< 60	161(41.0)	32(19.5)	193(34.6)
≥ 60	20(5.1)	2(1.2)	22(3.9)
DM in first−/second−/third-degree relatives	57(14.5)	28(17.1)	85(15.3)
Pancreatic diseases in first−/second−/third-degree relatives	12(3.1)	7(4.3)	19(3.4)
Elevated IgG^c^	16(4.1)	3(1.8)	19(3.4)
Elevated IgG4^c^	19(4.8)	2(1.2)	21(3.8)

*CP* Chronic pancreatitis, *ACP* Alcoholic chronic pancreatitis, *AIP* Autoimmune pancreatitis, *ICP* Idiopathic chronic pancreatitis, *DM* Diabetes mellitus, *PPC* Pancreatic pseudocyst, *SAP* Severe acute pancreatitis

^a^Mean ± SD

^b^123 patients with drinking history > 80 g/d included 112 cases with ACP, 7 cases with hereditary CP, 3 cases with anatomical abnormality and one case with post-traumatic CP

^c^Serum IgG and IgG4 were measured by immunoturbidimetry assay (Immage800 specific protein analyzer, Beckman, USA; BN2 specific protein analyzer, Siemens, Germany), and their upper limits were 15.6 g/l and 2.0 g/l respectively

### Comparison of frequency of autoantibodies between non-AIP CP patients and historial healthy controls

In this study, we selected autoantibodies with frequency > 0.5% (β2-GPI, SMA, ACL, AMA, anti SS-B, anti-ds DNA, anti-ss DNA, AHA, anti-RNP, anti-proteinase 3 IgG antibody) in non-AIP CP patients as search objects to compare and analyze, which were listed in Table 2. We identified 86 relevant citations through PubMed to be enrolled in this study (Additional file 1: Table S2). Then the frequency of these autoantibodies in non-AIP CP patients and historial healthy controls were calculated and compared respectively. χ^2^ or Fisher exact test results showed that the frequencies of serum β2-GPI and anti SS-B antibody in patients were significantly higher than that in historial healthy controls, and the frequencies of serum SMA and ACL antibody in patients were significantly lower than that in historial healthy controls (all *P* < 0.05).

**Table 2:** Comparison of positive rate of nine autoantibodies between non-AIP CP patients and historial healthy controls

Autoantibody	non-AIP CP patients			Historial Healthy controls			*P*
	Positive	Negative	Frequency (%)	Positive	Negative	Frequency (%)	
Anti-β2-glycoprotein I antibody	51	506	9.16	37	1839	1.97	< 0.001
Anti-smooth muscle antibody	17	540	3.05	24	302	7.36	0.003
Anticardiolipin antibody	16	541	2.87	93	1431	6.10	0.003
Anti-mitochondrial antibody	9	548	1.62	14	1340	1.03	0.224
Anti SS-B antibody	9	548	1.62	4	1659	0.24	< 0.001
Anti-double-stranded DNA antibody	8	549	1.44	0	202	0	0.190
Anti-single-stranded DNA antibody	6	551	1.08	14	568	2.41	0.088
Anti-histone antibody	5	552	0.90	14	711	1.93	0.129
Anti-ribonucleoprotein antibody	3	554	0.54	13	1078	1.19	0.201
Anti-proteinase 3 IgG antibody	3	554	0.54	0	237	0	0.558
Anti-PM-Scl antibody	2	555	0.36	–	–	–	–
Anti-neutrophil cytoplasmic antibody	2	555	0.36	–	–	–	–
Anti-ribosomal antibody	2	555	0.36	–	–	–	–
Anti SS-A antibody	1	556	0.18	–	–	–	–
Anti-Jo-1 antibody	1	556	0.18	–	–	–	–
Anti-SM antibody	1	556	0.18	–	–	–	–
Anti-myeloperoxidase antibody	1	556	0.18	–	–	–	–
anti-neutrophil perinuclear antibody	1	556	0.18	–	–	–	–
Anti-proliferating cell nuclear antigen antibody	1	556	0.18	–	–	–	–
Anti-Scl 70 antibody	0	556	0	–	–	–	–
Anti-nucleosome antibodies	0	556	0	–	–	–	–
Anti-glomerular basement membrane antibody	0	556	0	–	–	–	–

*CP* Chronic pancreatitis, *AIP* Autoimmune pancreatitis

### Related factors for positive β2-GPI antibody in non-AIP CP patients

As there were significant differences in frequency of serum β2-GPI, anti SS-B, SMA and ACL antibody between non-AIP CP patients and historial healthy controls, the relationship between these 4 autoantibodies and clinical characteristics were analyzed in non-AIP CP patients. The potential related factors were listed in Table 3 and were analyzed in the univariate analysis. As illustrated in Table 4, four variables showed a *P* value less than 0.15 in the univariate logistic regression analysis screening, and they were selected as candidates for multivariate logistic regression analysis. The result showed that diabetes mellitus (DM) in first−/second−/third-degree relatives (OR = 0.266, *P* = 0.033) was the protective factor of positive β2-GPI antibody while DM (OR = 2.768, *P* = 0.001) and common bile duct stricture (OR = 2.952, *P* = 0.007) were the risk factors. There were no related factors for other three differentially expressed autoantibodies (all *P* > 0.05), which were showed in (see Additional file 1: Table S3–S6).

**Table 3:** Related factors for positive β2-GPI antibody in non-AIP CP patients [n(%)]

Predictors	Positiven(*n* = 51)	Negetive(*n* = 506)	*P*
Female sex	15(29.4)	149(29.4)	0.996
BMI, kg/m^2^	21.49 ± 3.06^a^	25.23 ± 93.39	0.775
Age at the onset of CP, y^a^	40.31 ± 18.54^a^	36.22 ± 16.36	0.093
Age at the diagnosis of CP, y^a^	46.09 ± 15.33^a^	41.07 ± 15.15	0.135
Etiology			0.410
ACP	6(11.8)	106(20.9)	
ICP	41(80.4)	351(69.4)	
Abnormal anatomy of pancreatic duct	1(2.0)	28(5.5)	
Hereditary CP	3(5.9)	19(3.8)	
Post-traumatic CP	0(0)	2(0.4)	
Pancreatic stones	46(90.2)	470(92.9)	0.675
Adolescent	5(9.8)	67(13.2)	0.486
DM	22(43.1)	113(22.3)	0.001
Steatorrhea	14(27.5)	118(23.3)	0.508
Common bile duct stricture	11(19.6)	37(7.3)	0.006
PPC	8(15.7)	86(17.0)	0.812
SAP	2(3.9)	15(3.0)	1.000
Onset manifestations			0.110
Abdominal pain	35(68.6)	401(79.2)	
Pancreatic insufficiency	10(19.6)	78(15.4)	
Others	6(11.8)	27(5.3)	
Type of pain			0.576
None	7(13.7)	50(9.9)	
Repeat attacks of acute pancreatitis	14(27.5)	151(29.8)	
Repeat pain	21(41.2)	174(34.4)	
Repeat acute attacks and pain	7(13.7)	89(17.6)	
Chronic pain	2(3.9)	42(8.3)	
Drinking history (g/d)			0.078
0	38(74.5)	315(62.3)	
< 20	1(2.0)	17(3.4)	
20~80	5(9.8)	58(11.5)	
> 80	7(13.7)	116(22.9)	
Smoking history, pack-year			0.020
0	39(76.5)	303(59.9)	
< 60	11(21.6)	182(36.0)	
≥ 60	1(2.0)	21(4.2)	
DM in first−/second−/third-degree relatives	3(5.9)	82(16.2)	0.051
Pancreatic diseases in first−/second−/third-degree relatives	0(0)	19(3.8)	0.316
Elevated IgG^b^	3(5.9)	16(3.2)	0.538
Elevated IgG4^b^	5(9.8)	16(3.2)	0.047

*CP* Chronic pancreatitis, *ACP* Alcoholic chronic pancreatitis, *AIP* Autoimmune pancreatitis, *ICP* Idiopathic chronic pancreatitis, *DM* Diabetes mellitus, *PPC* Pancreatic pseudocyst, *SAP* Severe acute pancreatitis

^a^Mean ± SD

^b^Serum IgG and IgG4 were measured by immunoturbidimetry assay (Immage800 specific protein analyzer, Beckman, USA; BN2 specific protein analyzer, Siemens, Germany), and their upper limits were 15.6 g/l, 2.0 g/l respectively

**Table 4:** Related factors for positive β2-GPI antibody in non-AIP CP patients

Predictors	Univariate Analysis		Multivariate Analysis	
	OR(95% CI)	*P*	OR(95% CI)	*P*
Female sex	1.042(0.509–2.130)	0.911		
BMI, kg/m^2^	0.998(0.987–1.009)	0.751		
Age at the onset of CP, y^a^	0.992(0.954–1.031)	0.684		
Age at the diagnosis of CP, y^a^	1.028(0.986–1.071)	0.195		
Etiology		0.812		
ACP	Control			
ICP	0.991(0.059–16.668)	0.995		
Abnormal anatomy of pancreatic duct	0.377(0.013–11.052)	0.571		
Hereditary CP	1.770(0.164–19.055)	0.638		
Post-traumatic CP	0(0)	0.999		
Pancreatic stones	0.680(0.220–2.103)	0.503		
Adolescent	1.167(0.318–4.284)	0.816		
DM	2.315(1.174–4.566)	0.015	2.768(1.510–5.072)	0.001
Steatorrhea	1.462(0.705–3.033)	0.307		
Common bile duct stricture	2.785(1.159–6.693)	0.022	2.952(1.343–6.489)	0.007
PPC	0.938(0.397–2.215)	0.883		
SAP	0.906(0.160–5.139)	0.911		
Onset manifestations		0.240		
Abdominal pain	Control			
Pancreatic insufficiency	1.081(0.414–2.821)	0.874		
Others	2.949(0.780–11.145)	0.111		
Type of pain		0.879		
None	Control			
Repeat attacks of acute pancreatitis	1.131(0.292–4.377)	0.858		
Repeat pain	1.121(0.331–3.797)	0.854		
Repeat acute attacks and pain	1.132(0.245–5.243)	0.874		
Chronic pain	0.470(0.070–3.155)	0.437		
Drinking history (g/d)		0.828		
0	Control			
< 20	0.488(0.056–4.282)	0.517		
20~80	0.649(0.209–2.138)	0.497		
> 80	0.634(0.043–9.452)	0.741		
Smoking history, pack-year		0.445		
0	Control			
< 60	0.565(0.230–1.386)	0.213		
≥ 60	0.533(0.058–4.928)	0.579		
DM in first−/second−/third-degree relatives	0.272(0.076–0.076)	0.045	0.266(0.079–0.897)	0.033
Pancreatic diseases in first−/second−/third-degree relatives	0(0)	0.999		
Elevated IgG^b^	1.639(0.409–6.564)	0.485		
Elevated IgG4^b^	2.797(0.814–9.614)	0.103		

*CP* Chronic pancreatitis, *ACP* Alcoholic chronic pancreatitis, *AIP* Autoimmune pancreatitis; *ICP* Idiopathic chronic pancreatitis, *DM* Diabetes mellitus, *PPC* Pancreatic pseudocyst, *SAP* Severe acute pancreatitis

^a^Mean ± SD

^b^Serum IgG and IgG4 were measured by immunoturbidimetry assay (Immage800 specific protein analyzer, Beckman, USA; BN2 specific protein analyzer, Siemens, Germany), and their upper limits were 15.6 g/l, 2.0 g/l respectively

## Discussion

To our knowledge, the current study is the first study to compare the frequency of autoantibodies between non-AIP CP patients and historial healthy controls. This study totally detected 22 autoantibodies in 575 non-AIP CP patients after exclusion of patients combined with or newly diagnosed of other autoimmune diseases. Four autoantibodies (β2-GPI, anti SS-B, SMA and ACL antibody) were expressed differentially between non-AIP CP patients and historial healthy controls. DM in first−/second−/third-degree relatives was the protective factor of positive β2-GPI antibody while DM and common bile duct stricture were the risk factors. And there were no related factors for other three differentially expressed autoantibodies.

β2-GPI antibody, a major antigenic target for antiphospholipid antibodies, was the most frequent autoantibody in non-AIP CP patients. β2-GPI antibody could bine to negatively charged phospholipids and inhibit the coagulation cascade and platelet function [21]. Previous study had demonstrated that β2-GPI could interact with oxidized low density lipoprotein to form β2-GPI-ox-LDL complexes, and serum levels of β2-GPI-ox-LDL complexes were significantly elevated in autoimmune disorders, which may reliably help to predict the development of autoimmune-mediated atherosclerosis [22]. This present study showed that frequency of β2-GPI antibody in non-AIP CP patients was significantly higher than that in historial healthy controls (9.16% vs. 1.97%, *P* < 0.001). Multivariate logistic regression analysis result showed that DM and common bile duct stricture were risk factors of positive β2-GPI antibody while DM in first/second/third degree relatives was a protective factor. But there is no previous study to confirm the relationship between β2-GPI antibody and family history of DM, CBD stricture. Only a few studies have showed that β2-GPI antibody may participate in the occurrence and development of DM [23]. Cojocaru et al. [24] indicated that the positive rate of anti-IgG β2-GPI in type 2 diabetes mellitus patients with diabetic retinopathy (DR) was significantly higher than that in patients without DR (85% vs. 21%, RR 4.640). However, Tarkun et al. [25] found that there was no significant association between β2-GPI antibody and vascular complications in type 2 diabetes mellitus patients, so β2-GPI antibody may not have a major role in the pathogenesis of diabetic complications in type 2 diabetes mellitus patients. In brief, it’s unclear whether β2-GPI antibody was related to the occurrence and development of clinical events of CP until now.

At present, CP is regarded as a disease with multiple etiological factors including alcohol, autoimmunity, biliary tract diseases, etc. And alcohol and autoimmunity factors may coexist. In the past, patients suspected of CP admitted to our center for the first time would be received history taking, physical examination, imaging examination and laboratory examination (including serum autoantibody detection) for diagnosis, etiology identification, treatment guidance and prognosis evaluation. The present study determined four differentially expressed autoantibodies between non-AIP CP patients and historial healthy controls, among them β2-GPI antibody were expressed most frequently. As β2-GPI antibody was independently associated with DM and common bile duct stricture, it may be a potential serum marker to predict the occurrence of these clinical events. Although previous studies found several serum markers for early diagnosis [26] and differential diagnosis [27] of CP, they were not widely used in clinic practice. Until now, the diagnosis of CP is mainly based on the clinical manifestation and imaging findings. Although this current study identified four differentially expressed autoantibodies between non-AIP CP patients and historial healthy controls, they had limited value in diagnosing non-AIP CP, and could not help the differentiation of non-AIP CP from other pancreatic diseases including pancreatic cancer, intraductal papillary mucinous neoplasm of the pancreas, cystic pancreatic lesions and so on because of low specificity [28]. And there is no research to confirm that these four autoantibodies are protective against development of CP up to now. Therefore, we don’t recommend that autoantibodies test should be a conventional examination for diagnosis and differential diagnosis of CP unless suspected of AIP, whose several diagnostic criteria [7–9] had include presence of autoantibodies. Another recent study in our center showed that the positive rates of antinuclear antibody, anti-SSA antibody, and anti-SSB antibody of patients with AIP were 17.1, 11.4 and 8.6% respectively, and then we considered that autoantibody could be a subsidiary indicator for the diagnosis of AIP [29]. Treatment options of CP mainly include pancreatic enzyme replacement therapy, insulin infusion injection, endoscopic therapy and surgery, which were mostly determined by clinical symptom, blood sugar level, imaging findings and patients’ subjective will. Although the presence of autoantibodies were related with DM and common bile duct stricture in this present study, they could not be served as predictors for the clinical events, and had little significance in guiding clinical treatments and evaluating curative effect until now.

There are several limitations of the present study. Firstly, the observational study design (cohort study) is inherent to selection bias. And we chose control group through literature search in PubMed due to difficulty for large-scale collecting the serum of healthy people to detect all antibodies in clinical, but the historial healthy controls may increase the inaccuracy of target autoantibodies’ frequencies as there may be differences among different races, countries, regions and nations. Secondly, this study lack of estimate on causality between autoantibody and CP occurrence as the samples were tested once disease had occurred. Thirdly, this current study belongs to cross-sectional study, so it is not clearly whether autoantibody test could predict the occurrence of clinical events and treatment prognosis of CP.

## Conclusions

Four autoantibodies were expressed differentially between non-AIP CP patients and historial healthy controls. Due to limited significance for diagnosis and treatment of CP, autoantibodies detection is not recommended conventionally unless suspected of AIP.

## Additional file

Additional file 1: **Table S1.** Sensitivity and specificity of test method of 22 autoantibodies. Peripheral venous blood samples were obtained from all enrolled patients at admission to detect 22 common autoantibodies (EUROIMMUN Medical Laboratory Diagnostics Stock Company, Lübeck, German), whose sensitivity and specificity were provided in Additional file 1: Table S1. **Table S2.** Search strategy and result in PubMed (up until May 4th, 2017) In order to compare the frequency of autoantibodies between non-AIP CP patients and healthy controls, a literature search for autoantibodies with frequency > 0.5% was performed through PubMed to identify eligible studies published. These search strategy and result are provided in Additional file 1: Table S2. **Table S3.** Univariate analysis for smooth muscle antibody, anticardiolipin antibody and anti SS-B antibody in non-AIP CP patients [n(%)]. Univariate analysis result showed that there were no significant differences in all clinical data between patients with positive and negative other three differentially expressed autoantibodies (smooth muscle antibody, anticardiolipin antibody and anti SS-B antibody) (all *P* > 0.05). **Table S4.** Related factors for positive smooth muscle antibody in non-AIP CP patients. Univariate logistic regression analysis result showed that there were no related factors for positive smooth muscle antibody in non-AIP CP patients (*P* > 0.05). **Table S5.** Related factors for positive anticardiolipin antibody in non-AIP CP patients. Univariate logistic regression analysis result showed that there were no related factors for positive anticardiolipin antibody in non-AIP CP patients (*P* > 0.05). **Table S6.** Related factors for positive anti SS-B antibody in non-AIP CP patients. Univariate logistic regression analysis result showed that there were no related factors for positive anti SS-B antibody in non-AIP CP patients (*P* > 0.05). (DOCX 165 kb)

## Abbreviations

ACL: Anticardiolipin
ACP: Alcoholic chronic pancreatitis
AHA: Anti-histone antibody
AIP: Autoimmune pancreatitis
AMA: Anti-mitochondrial antibody
Anti-ds DNA: Anti-double-stranded DNA
Anti-RNP: Anti-ribonucleoprotein
Anti-ss DNA: Anti-single-stranded DNA
CI: Confidence interval
CP: Chronic pancreatitis
DM: Diabetes mellitus
DR: Diabetic retinopathy
ICP: Idiopathic chronic pancreatitis
OR: Odds ratio
PPC: Pancreatic pseudocyst
RA: Rheumatoid arthritis
SAP: Severe acute pancreatitis
SD: Standard deviation
SMA: Anti-smooth muscle antibody
β2-GPI: Anti-β2-glycoprotein I
