# Supplementary material for: Autoantibody detection is not recommended for chronic pancreatitis: a cross-sectional Study of 557 patients
Source: BMC Gastroenterol. 2019 Feb 14;19:31. doi: 10.1186/s12876-019-0947-7 (PMC6376735; doi:10.1186/s12876-019-0947-7)
Supplement: Supplementary file 1 — Table S1. Sensitivity and specificity of test method of 22 autoantibodies. Peripheral venous blood samples were obtained from all enrolled patients at admission to detect 22 common autoantibodies (EUROIMMUN Medical Laboratory Diagnostics Stock Company, Lübeck, German), whose sensitivity and specificity were provided in Additional file 1: Table S1. Table S2. Search strategy and result in PubMed (up until May 4th, 2017) In order to compare the frequency of autoantibodies between non-AIP CP patients and healthy controls, a literature search for autoantibodies with frequency > 0.5% was performed through PubMed to identify eligible studies published. These search strategy and result are provided in Additional file 1: Table S2. Table S3. Univariate analysis for smooth muscle antibody, anticardiolipin antibody and anti SS-B antibody in non-AIP CP patients [n(%)]. Univariate analysis result showed that there were no significant differences in all clinical data between patients with positive and negative other three differentially expressed autoantibodies (smooth muscle antibody, anticardiolipin antibody and anti SS-B antibody) (all P > 0.05). Table S4. Related factors for positive smooth muscle antibody in non-AIP CP patients. Univariate logistic regression analysis result showed that there were no related factors for positive smooth muscle antibody in non-AIP CP patients (P > 0.05). Table S5. Related factors for positive anticardiolipin antibody in non-AIP CP patients. Univariate logistic regression analysis result showed that there were no related factors for positive anticardiolipin antibody in non-AIP CP patients (P > 0.05). Table S6. Related factors for positive anti SS-B antibody in non-AIP CP patients. Univariate logistic regression analysis result showed that there were no related factors for positive anti SS-B antibody in non-AIP CP patients (P > 0.05). (DOCX 165 kb) [file 12876_2019_947_MOESM1_ESM.docx]

**Additional file 1**

**Table S1. Sensitivity and specificity of test method of 22 autoantibodies**

| Autoantibody | Test method | Sensitivity (%) | Specificity (%) |
| --- | --- | --- | --- |
| Anti-double-stranded DNA antibody | EUROLINE | 94.0 | 100 |
| Anti-single-stranded DNA antibody | EUROLINE | 100 | 100 |
| Anti-SM antibody | EUROLINE | 100 | 100 |
| Anti-ribonucleoprotein antibody | EUROLINE | 100 | 96.0 |
| Anti SS-A antibody | EUROLINE | 100 | 95.0 |
| Anti SS-B antibody | EUROLINE | 100 | 100 |
| Anti-Jo-1 antibody | EUROLINE | 100 | 100 |
| Anti-Scl 70 antibody | EUROLINE | 100 | 100 |
| Anti-proliferating cell nuclear antigen antibody | EUROLINE | 100 | 99.0 |
| Anti-nucleosome antibodies | EUROLINE | 97.0 | 100 |
| Anti-histone antibody | EUROLINE | 78.0 | 97.0 |
| Anti-ribosomal antibody | EUROLINE | 82.0 | 100 |
| Anti-PM-Scl antibody | EUROLINE | 100 | 99.0 |
| Anti-smooth muscle antibody | Indirect immunofluorescence | 100 | 100 |
| Anti-mitochondrial antibody | Indirect immunofluorescence | 100 | 100 |
| Anti-neutrophil cytoplasmic antibody | Indirect immunofluorescence | 80.0 | 99.0 |
| Anti-neutrophil perinuclear antibody | Indirect immunofluorescence | 100 | 99.0 |
| Anti-glomerular basement membrane antibody | Enzyme-linked immunosorbent assay | 99.0 | 100 |
| Anti-proteinase 3 IgG antibody | Enzyme-linked immunosorbent assay | 94.0 | 99.0 |
| Anti-myeloperoxidase antibody | Enzyme-linked immunosorbent assay | 93.3 | 99.8 |
| Anti-β2-glycoprotein I antibody | Enzyme-linked immunosorbent assay | 86.0 | 96.8 |
| Anticardiolipin antibody | Enzyme-linked immunosorbent assay | 81.0 | 99.2 |

**Table S2. Search strategy and result in PubMed (up until May 4^th^, 2017)**

| Autoantibody | Search strategy in PubMed | identified articles | Articles enrolled for study finally |
| --- | --- | --- | --- |
| Anti–β2-glycoprotein I antibody | ((((((("beta 2-glycoprotein i"[MeSH Terms] OR "beta 2-glycoprotein i"[All Fields] OR "beta2 glycoprotein i"[All Fields]) AND ("antibodies"[MeSH Terms] OR "antibodies"[All Fields])) OR (anti-beta2-glycoprotein[All Fields] AND 1[All Fields])) OR anti-beta2GP1[All Fields]) OR (anti-beta2[All Fields] AND ("glycoproteins"[MeSH Terms] OR "glycoproteins"[All Fields] OR "glycoprotein"[All Fields]))) OR beta2-GPI[All Fields]) OR abeta2GPI[All Fields]) AND ((((("Cohort Studies"[Mesh] OR "Case-Control Studies"[Mesh]) OR "Cross-Sectional Studies"[Mesh]) OR ("cohort studies"[MeSH Terms] OR ("cohort"[All Fields] AND "studies"[All Fields]) OR "cohort studies"[All Fields])) OR ("case-control studies"[MeSH Terms] OR ("case-control"[All Fields] AND "studies"[All Fields]) OR "case-control studies"[All Fields] OR ("case"[All Fields] AND "control"[All Fields] AND "studies"[All Fields]) OR "case control studies"[All Fields])) OR ("cross-sectional studies"[MeSH Terms] OR ("cross-sectional"[All Fields] AND "studies"[All Fields]) OR "cross-sectional studies"[All Fields] OR ("cross"[All Fields] AND "sectional"[All Fields] AND "studies"[All Fields]) OR "cross sectional studies"[All Fields])) | 471 | 25[[1-25](#_ENREF_1)] |
| Smooth muscle antibody | ((((("Cohort Studies"[Mesh] OR "Case-Control Studies"[Mesh]) OR "Cross-Sectional Studies"[Mesh]) OR ("cohort studies"[MeSH Terms] OR ("cohort"[All Fields] AND "studies"[All Fields]) OR "cohort studies"[All Fields])) OR ("case-control studies"[MeSH Terms] OR ("case-control"[All Fields] AND "studies"[All Fields]) OR "case-control studies"[All Fields] OR ("case"[All Fields] AND "control"[All Fields] AND "studies"[All Fields]) OR "case control studies"[All Fields])) OR ("cross-sectional studies"[MeSH Terms] OR ("cross-sectional"[All Fields] AND "studies"[All Fields]) OR "cross-sectional studies"[All Fields] OR ("cross"[All Fields] AND "sectional"[All Fields] AND "studies"[All Fields]) OR "cross sectional studies"[All Fields])) AND (“smooth muscle antibody”[All Fields] OR “anti-smooth muscle antibody”[All Fields]) | 43 | 2[[26](#_ENREF_26),[27](#_ENREF_27)] |
| Anticardiolipin antibody | (((((("Cohort Studies"[Mesh] OR "Case-Control Studies"[Mesh]) OR "Cross-Sectional Studies"[Mesh]) OR ("cohort studies"[MeSH Terms] OR ("cohort"[All Fields] AND "studies"[All Fields]) OR "cohort studies"[All Fields])) OR ("case-control studies"[MeSH Terms] OR ("case-control"[All Fields] AND "studies"[All Fields]) OR "case-control studies"[All Fields] OR ("case"[All Fields] AND "control"[All Fields] AND "studies"[All Fields]) OR "case control studies"[All Fields])) OR ("cross-sectional studies"[MeSH Terms] OR ("cross-sectional"[All Fields] AND "studies"[All Fields]) OR "cross-sectional studies"[All Fields] OR ("cross"[All Fields] AND "sectional"[All Fields] AND "studies"[All Fields]) OR "cross sectional studies"[All Fields])) AND (anticardiolipin[Title/Abstract])) AND ((control[Title/Abstract] OR controls[Title/Abstract]) AND healthy[Title/Abstract]) | 171 | 24[[7](#_ENREF_7),[12](#_ENREF_12),[15](#_ENREF_15),[16](#_ENREF_16),[28-47](#_ENREF_28)] |
| Anti-mitochondrial antibody | ((((("Cohort Studies"[Mesh] OR "Case-Control Studies"[Mesh]) OR "Cross-Sectional Studies"[Mesh]) OR ("cohort studies"[MeSH Terms] OR ("cohort"[All Fields] AND "studies"[All Fields]) OR "cohort studies"[All Fields])) OR ("case-control studies"[MeSH Terms] OR ("case-control"[All Fields] AND "studies"[All Fields]) OR "case-control studies"[All Fields] OR ("case"[All Fields] AND "control"[All Fields] AND "studies"[All Fields]) OR "case control studies"[All Fields])) OR ("cross-sectional studies"[MeSH Terms] OR ("cross-sectional"[All Fields] AND "studies"[All Fields]) OR "cross-sectional studies"[All Fields] OR ("cross"[All Fields] AND "sectional"[All Fields] AND "studies"[All Fields]) OR "cross sectional studies"[All Fields])) AND (“antimitochondrial antibody”[All Fields] OR “anti-mitochondrial antibody”[All Fields]) | 114 | 4[[27](#_ENREF_27),[48-50](#_ENREF_48)] |
| Anti SS-B antibody | ((((("Cohort Studies"[Mesh] OR "Case-Control Studies"[Mesh]) OR "Cross-Sectional Studies"[Mesh]) OR ("cohort studies"[MeSH Terms] OR ("cohort"[All Fields] AND "studies"[All Fields]) OR "cohort studies"[All Fields])) OR ("case-control studies"[MeSH Terms] OR ("case-control"[All Fields] AND "studies"[All Fields]) OR "case-control studies"[All Fields] OR ("case"[All Fields] AND "control"[All Fields] AND "studies"[All Fields]) OR "case control studies"[All Fields])) OR ("cross-sectional studies"[MeSH Terms] OR ("cross-sectional"[All Fields] AND "studies"[All Fields]) OR "cross-sectional studies"[All Fields] OR ("cross"[All Fields] AND "sectional"[All Fields] AND "studies"[All Fields]) OR "cross sectional studies"[All Fields])) AND (“SS-B”[Title/Abstract]) | 97 | 5[[49](#_ENREF_49),[51-54](#_ENREF_51)] |
| Anti-double-stranded DNA antibody | ((((("Cohort Studies"[Mesh] OR "Case-Control Studies"[Mesh]) OR "Cross-Sectional Studies"[Mesh]) OR ("cohort studies"[MeSH Terms] OR ("cohort"[All Fields] AND "studies"[All Fields]) OR "cohort studies"[All Fields])) OR ("case-control studies"[MeSH Terms] OR ("case-control"[All Fields] AND "studies"[All Fields]) OR "case-control studies"[All Fields] OR ("case"[All Fields] AND "control"[All Fields] AND "studies"[All Fields]) OR "case control studies"[All Fields])) OR ("cross-sectional studies"[MeSH Terms] OR ("cross-sectional"[All Fields] AND "studies"[All Fields]) OR "cross-sectional studies"[All Fields] OR ("cross"[All Fields] AND "sectional"[All Fields] AND "studies"[All Fields]) OR "cross sectional studies"[All Fields])) AND (“ds-DNA” [All Fields]) | 105 | 5[[55-59](#_ENREF_55)] |
| Anti-single-stranded DNA antibody | ((“ss-DNA” [Title/Abstract]) AND ((control[Title/Abstract] OR controls[Title/Abstract]) OR healthy[Title/Abstract])) | 102 | 5[[59-63](#_ENREF_59)] |
| Anti-histone antibody | (anti-histone[Title/Abstract] OR antihistone[Title/Abstract]) AND ((control[Title/Abstract] OR controls[Title/Abstract]) OR healthy[Title/Abstract]) | 146 | 10[[64-73](#_ENREF_64)] |
| Ribonucleoprotein antibody | ((RNP [Title/Abstract] OR Ribonucleoprotein [Title/Abstract]) AND antibody[Title/Abstract]) AND ((control[Title/Abstract] OR controls[Title/Abstract]) OR healthy[Title/Abstract]) | 205 | 9[[49](#_ENREF_49),[68](#_ENREF_68),[74-80](#_ENREF_74)] |
| Anti-proteinase 3 IgG antibody | (antiproteinase 3 [Title/Abstract] OR anti-proteinase 3 [Title/Abstract]) AND ((control[Title/Abstract] OR controls[Title/Abstract]) OR healthy[Title/Abstract]) | 42 | 6[[81-86](#_ENREF_81)] |

**Table S3** **Univariate analysis for smooth muscle antibody, anticardiolipin antibody and anti SS-B antibody in non-AIP CP patients [n(%)]**

| Predictors | Smooth muscle antibody | | | Anticardiolipin antibody | | | Anti SS-B antibody | | |
| --- | --- | --- | --- | --- | --- | --- | --- | --- | --- |
|  | Positive(n=17) | Negative(n=540) | *P* | Positive(n=16) | Negative(n=541) | *P* | Positive(n=9) | Negative(n=548) | *P* |
| Female sex | 8(47.1) | 156(28.9) | 0.106 | 4(25.0) | 16(29.6) | 0.907 | 3(33.3) | 161(29.4) | 1.000 |
| BMI, kg/m^2^ | 22.28±3.78* | 24.97±90.40* | 0.902 | 22.03±3.67* | 24.98±90.32* | 0.896 | 21.21±3.00* | 24.95±89.74* | 0.901 |
| Age at the onset of CP, y* | 36.17±18.07* | 36.61±16.56* | 0.915 | 39.74±15.95* | 36.50±16.62* | 0.442 | 34.38±22.37* | 36.63±16.51* | 0.686 |
| Age at the diagnosis of CP, y* | 41.52±15.22* | 41.53±15.23* | 0.997 | 42.79±13.66* | 41.49±15.27* | 0.738 | 41.69±14.88* | 41.53±15.24* | 0.975 |
| Etiology |  |  | 0.928 |  |  | 0.762 |  |  | 0.695 |
| ACP | 4(23.5) | 108(20.0) |  | 3(18.8) | 109(20.1) |  | 1(11.1) | 111(20.3) |  |
| ICP | 12(70.6) | 380(70.4) |  | 13(81.3) | 379(70.1) |  | 6(66.7) | 386(70.4) |  |
| Abnormal anatomy of pancreatic duct | 1(5.9) | 28(5.2) |  | 0(0) | 29(5.4) |  | 1(11.1) | 28(5.1) |  |
| Hereditary CP | 0(0) | 22(4.1) |  | 0(0) | 22(4.1) |  | 1(11.1) | 21(3.8) |  |
| Post-traumatic CP | 0(0) | 2(0.4) |  | 0(0) | 2(0.4) |  | 0(0) | 2(0.4) |  |
| Pancreatic stones | 15(88.2) | 501(92.8) | 0.815 | 14(87.5) | 502(92.8) | 0.754 | 9(100.0) | 507(92.5) | 1.000 |
| Adolescent | 1(5.9) | 71(13.1) | 0.609 | 1(6.3) | 71(13.1) | 0.667 | 2(22.2) | 70(12.8) | 0.736 |
| DM | 3(17.6) | 132(24.4) | 0.721 | 3(18.8) | 132(24.4) | 0.823 | 2(22.2) | 133(24.3) | 1.000 |
| Steatorrhea | 2(11.8) | 130(24.1) | 0.376 | 2(12.5) | 130(24.0) | 0.441 | 4(44.4) | 127(23.2) | 0.061 |
| Common bile duct stricture | 1(5.9) | 46(8.5) | 1.000 | 3(18.8) | 44(8.1) | 0.294 | 1(11.1) | 45(8.2) | 0.172 |
| PPC | 3(17.6) | 91(16.9) | 1.000 | 4(25.0) | 90(16.6) | 0.588 | 2(22.2) | 92(16.8) | 1.000 |
| SAP | 1(5.9) | 16(3.0) | 0.414 | 1(6.3) | 16(3.0) | 0.395 | 1(11.1) | 16(2.9) | 0.245 |
| Onset manifestations |  |  | 0.573 |  |  | 0.476 |  |  | 0.670 |
| Abdominal pain | 14(82.4) | 422(78.1) |  | 11(68.8) | 425(78.6) |  | 8(88.9) | 428(78.1) |  |
| Pancreatic insufficiency | 3(17.6) | 85(15.7) |  | 3(18.8) | 85(15.7) |  | 1(11.1) | 87(15.9) |  |
| Others | 0(0) | 33(6.1) |  | 2(12.5) | 31(5.7) |  | 0(0) | 33(6.0) |  |
| Type of pain |  |  | 0.306 |  |  | 0.414 |  |  | 0.072 |
| None | 1(5.9) | 56(10.4) |  | 1(5.9) | 56(10.4) |  | 1(11.1) | 56(10.2) |  |
| Repeat attacks of acute pancreatitis | 4(23.5) | 161(29.8) |  | 3(18.8) | 162(29.9) |  | 2(22.2) | 163(29.7) |  |
| Repeat pain | 4(23.5) | 191(35.4) |  | 7(43.8) | 188(34.8) |  | 1(11.1) | 194(35.4) |  |
| Repeat acute attacks and pain | 5(29.4) | 91(16.9) |  | 2(12.5) | 94(17.4) |  | 5(55.6) | 91(16.6) |  |
| Chronic pain | 3(17.6) | 41(7.6) |  | 3(18.8) | 41(7.6) |  | 0(0) | 44(8.0) |  |
| Drinking history (g/d) |  |  | 0.804 |  |  | 0.409 |  |  | 0.720 |
| 0 | 10(58.8) | 343(63.5) |  | 12(75.0) | 341(63.0) |  | 6(66.7) | 347(63.3) |  |
| <20 | 2(11.8) | 16(3.0) |  | 0(0) | 18(3.3) |  | 0(0) | 18(3.3) |  |
| 20~80 | 1(5.9) | 62(11.5) |  | 1(5.9) | 62(11.5) |  | 2(22.2) | 61(11.1) |  |
| >80 | 4(23.5) | 119(22.0) |  | 3(18.8) | 120(22.2) |  | 1(11.1) | 122(22.3) |  |
| Smoking history, pack-year |  |  | 0.451 |  |  | 0.479 |  |  | 0.802 |
| 0 | 9(52.9) | 333(61.7) |  | 11(68.8) | 331(61.2) |  | 5(55.6) | 337(61.5) |  |
| <60 | 7(41.2) | 186(34.4) |  | 5(31.3) | 188(34.8) |  | 4(44.4) | 189(34.5) |  |
| ≥60 | 1(4.5) | 21(3.9) |  | 0(0) | 22(4.1) |  | 0(0) | 22(4.0) |  |
| DM in first-/second-/third-degree relatives | 4(23.5) | 81(15.0) | 0.535 | 1(6.3) | 84(15.5) | 0.507 | 1(11.1) | 84(15.3) | 1.000 |
| Pancreatic diseases in first-/second-/third-degree relatives | 1(5.9) | 18(3.3) | 0.451 | 0(0) | 19(3.5) | 1.000 | 0(0) | 19(3.5) | 1.000 |
| Elevated IgG^#^ | 0(0) | 19(3.5) | 1.000 | 0(0) | 19(3.5) | 1.000 | 1(11.1) | 18(3.3) | 0.270 |
| Elevated IgG4^#^ | 1(5.9) | 20(3.7) | 0.485 | 1(5.9) | 20(3.7) | 0.464 | 0(0) | 21(3.8) | 1.000 |

CP = chronic pancreatitis; ACP = alcoholic chronic pancreatitis; AIP = autoimmune pancreatitis; ICP = idiopathic chronic pancreatitis; DM = diabetes mellitus; PPC = pancreatic pseudocyst; SAP = severe acute pancreatitis.

*Mean ± SD.

^#^Serum IgG and IgG4 were measured by immunoturbidimetry assay (Immage800 specific protein analyzer, Beckman, USA; BN2 specific protein analyzer, Siemens, Germany), and their upper limits were 15.6g/l, 2.0g/l respectively.

**Table S4. Related factors for positive smooth muscle antibody in non-AIP CP patients**

| Predictors | Univariate Analysis | |
| --- | --- | --- |
|  | OR(95% CI) | *P* |
| Female sex | 3.266(0.987-10.808) | 0.053 |
| BMI, kg/m^2^ | 1.081(0.941-1.241) | 0.271 |
| Age at the onset of CP, y* | 0.991(0.927-1.059) | 0.786 |
| Age at the diagnosis of CP, y* | 0.988(0.922-1.059) | 0.737 |
| Etiology |  | 0.999 |
| ACP | Control | |
| ICP | 0.853(0.270-2.697) | 0.786 |
| Abnormal anatomy of pancreatic duct | 0.964(0.104-8.971) | 0.975 |
| Hereditary CP | 0(0) | 0.999 |
| Post-traumatic CP | 0(0) | 0.999 |
| Pancreatic stones | 0.427(0.068-2.675) | 0.364 |
| Adolescent | 0.151(0.012-1.890) | 0.143 |
| DM | 0.684(0.157-2.984) | 0.614 |
| Steatorrhea | 0.312(0.063-1.543) | 0.153 |
| Common bile duct stricture | 0.763(0.084-6.945) | 0.810 |
| PPC | 0.817(0.177-3.784) | 0.796 |
| SAP | 1.731(0.137-21.862) | 0.671 |
| Onset manifestations |  | 0.784 |
| Abdominal pain | Control | |
| Pancreatic insufficiency | 1.931(0.302-12.327) | 0.487 |
| Others | 0(0) | 0.955 |
| Type of pain |  | 0.209 |
| None | Control | |
| Repeat attacks of acute pancreatitis | 1.366(0.084-22.265) | 0.827 |
| Repeat pain | 1.233(0.080-18..924) | 0.881 |
| Repeat acute attacks and pain | 4.068(0.217-76.124) | 0.348 |
| Chronic pain | 6.304(0.356-111.593) | 0.209 |
| Drinking history (g/d) |  | 0.285 |
| 0 | Control | |
| <20 | 4.288(0.867-21.211) | 0.074 |
| 20~80 | 0.553(0.070-4.399) | 0.576 |
| >80 | 1.153(0.355-3.745) | 0.813 |
| Smoking history, pack-year |  | 0.291 |
| 0 | Control | |
| <60 | 2.958(0.718-12.183) | 0.133 |
| ≥60 | 3.647(0.292-45.526) | 0.315 |
| DM in first-/second-/third-degree relatives | 1.415(0.392-5.113) | 0.596 |
| Pancreatic diseases in first-/second-/third-degree relatives | 1.950(0.204-18.605) | 0.562 |
| Elevated IgG^#^ | 0(0) | 0.998 |
| Elevated IgG4^#^ | 2.155(0.215-21.621) | 0.514 |

CP = chronic pancreatitis; ACP = alcoholic chronic pancreatitis; ICP = idiopathic chronic pancreatitis; DM = diabetes mellitus; PPC = pancreatic pseudocyst; SAP = severe acute pancreatitis.

*Mean ± SD.

^#^Serum IgG and IgG4 were measured by immunoturbidimetry assay (Immage800 specific protein analyzer, Beckman, USA; BN2 specific protein analyzer, Siemens, Germany), and their upper limits were 15.6g/l and 2.0g/l respectively.

**Table S5. Related factors for positive anticardiolipin antibody in non-AIP CP patients**

| Predictors | Univariate Analysis | |
| --- | --- | --- |
|  | OR(95% CI) | *P* |
| Female sex | 0.992(0.280-3.513) | 0.990 |
| BMI, kg/m^2^ | 0.999(0.989-1.010) | 0.877 |
| Age at the onset of CP, y* | 1.029(0.950-1.115) | 0.488 |
| Age at the diagnosis of CP, y* | 0.961(0.883-1.045) | 0.351 |
| Etiology |  | 0.998 |
| ACP | Control | |
| ICP | 1.246(0.349-4.453) | 0.735 |
| Abnormal anatomy of pancreatic duct | 0(0) | 0.998 |
| Hereditary CP | 0(0) | 0.998 |
| Post-traumatic CP | 0(0) | 0.998 |
| Pancreatic stones | 0.790(0.139-4.496) | 0.790 |
| Adolescent | 0.376(0.030-4.748) | 0.449 |
| DM | 0.714(0.162-3.140) | 0.656 |
| Steatorrhea | 0.367(0.070-1.940) | 0.238 |
| Common bile duct stricture | 2.585(0.622-10.736) | 0.191 |
| PPC | 1.402(0.398-4.941) | 0.599 |
| SAP | 3.013(0.294-30.869) | 0.353 |
| Onset manifestations |  | 0.114 |
| Abdominal pain | Control | |
| Pancreatic insufficiency | 2.693(0.575-12.612) | 0.209 |
| Others | 6.943(1.004-48.000) | 0.050 |
| Type of pain |  | 0.217 |
| None | Control | |
| Repeat attacks of acute pancreatitis | 3.356(0.229-49.200) | 0.377 |
| Repeat pain | 5.246(0.456-60.395) | 0.184 |
| Repeat acute attacks and pain | 5.384(0.276-104.998) | 0.267 |
| Chronic pain | 18.904(1.158-308.578) | 0.039 |
| Drinking history (g/d) |  | 0.864 |
| 0 | Control | |
| <20 | 0(0) | 0.998 |
| 20~80 | 0.458(0.059-3.588) | 0.457 |
| >80 | 0.710(0.197-2.561) | 0.601 |
| Smoking history, pack-year |  | 0.999 |
| 0 | Control | |
| <60 | 0.975(0.231-4.122) | 0.972 |
| ≥60 | 0(0) | 0.998 |
| DM in first-/second-/third-degree relatives | 0.386(0.046-3.208) | 0.378 |
| Pancreatic diseases in first-/second-/third-degree relatives | 0(0) | 0.998 |
| Elevated IgG^#^ | 0(0) | 0.998 |
| Elevated IgG4^#^ | 1.389(0.137-14.111) | 0.781 |

CP = chronic pancreatitis; ACP = alcoholic chronic pancreatitis; ICP = idiopathic chronic pancreatitis; DM = diabetes mellitus; PPC = pancreatic pseudocyst; SAP = severe acute pancreatitis.

*Mean ± SD.

^#^Serum IgG and IgG4 were measured by immunoturbidimetry assay (Immage800 specific protein analyzer, Beckman, USA; BN2 specific protein analyzer, Siemens, Germany), and their upper limits were 15.6g/l and 2.0g/l respectively.

**Table S6. Related factors for positive anti SS-B antibody in non-AIP CP patients**

| Predictors | Univariate Analysis | |
| --- | --- | --- |
|  | OR(95% CI) | *P* |
| Female sex | 0.764(0.132-4.415) | 0.764 |
| BMI, kg/m^2^ | 1.008(0.806-1.260) | 0.947 |
| Age at the onset of CP, y* | 0.958(0.855-1.074) | 0.464 |
| Age at the diagnosis of CP, y* | 1.062(0.941-1.199) | 0.328 |
| Etiology |  | 0.743 |
| ACP | Control | |
| ICP | 1.725(0.206-14.483) | 0.615 |
| Abnormal anatomy of pancreatic duct | 3.964(0.240-65.363) | 0.335 |
| Hereditary CP | 5.286(0.318-87.863) | 0.246 |
| Post-traumatic CP | 0(0) | 0.998 |
| Pancreatic stones | 0(0) | 0.998 |
| Adolescent | 1.139(0.063-20.744) | 0.930 |
| DM | 0.445(0.065-3.040) | 0.409 |
| Steatorrhea | 2.652(0.702-10.024) | 0.151 |
| Common bile duct stricture | 1.364(0.167-11.147) | 0.772 |
| PPC | 1.497(0.227-9.885) | 0.675 |
| SAP | 5.622(0.278-113.797) | 0.261 |
| Onset manifestations |  | 0.901 |
| Abdominal pain | Control | |
| Pancreatic insufficiency | 0.615(0.076-4.980) | 0.649 |
| Others | 0(0) | 0.998 |
| Type of pain |  | 0.164 |
| None | Control | |
| Repeat attacks of acute pancreatitis | 0.687(0.061-7.724) | 0.761 |
| Repeat pain | 0.289(0.018-4.6894) | 0.382 |
| Repeat acute attacks and pain | 3.077(0.350-27.021) | 0.311 |
| Chronic pain | 0(0) | 0.998 |
| Drinking history (g/d) |  | 0.725 |
| 0 | Control | |
| <20 | 0(0) | 0.998 |
| 20~80 | 1.896(0.374-9.613) | 0.440 |
| >80 | 0.474(0.057-3.977) | 0.492 |
| Smoking history, pack-year |  | 0.842 |
| 0 | Control | |
| <60 | 1.883(0.228-15.562) | 0.557 |
| ≥60 | 0(0) | 0.998 |
| DM in first-/second-/third-degree relatives | 0.526(0.049-5.613) | 0.594 |
| Pancreatic diseases in first-/second-/third-degree relatives | 0(0) | 0.998 |
| Elevated IgG^#^ | 12.347(0.449-339.482) | 0.137 |
| Elevated IgG4^#^ | 0(0) | 0.998 |

CP = chronic pancreatitis; ACP = alcoholic chronic pancreatitis; ICP = idiopathic chronic pancreatitis; DM = diabetes mellitus; PPC = pancreatic pseudocyst; SAP = severe acute pancreatitis.

*Mean ± SD.

^#^Serum IgG and IgG4 were measured by immunoturbidimetry assay (Immage800 specific protein analyzer, Beckman, USA; BN2 specific protein analyzer, Siemens, Germany), and their upper limits were 15.6g/l and 2.0g/l respectively.

**References**

1. Roldan V, Marin F, Pineda J, Marco P, Corral J, et al. (2002) [Annexin V levels in survivors of early myocardial infarction]. Rev Esp Cardiol 55: 1230-1234.

2. Previtali S, Barbui T, Galli M (2002) Anti-beta2-glycoprotein I and anti-prothrombin antibodies in antiphospholipid-negative patients with thrombosis: a case control study. Thromb Haemost 88: 729-732.

3. Faden D, Tincani A, Tanzi P, Spatola L, Lojacono A, et al. (1997) Anti-beta 2 glycoprotein I antibodies in a general obstetric population: preliminary results on the prevalence and correlation with pregnancy outcome. Anti-beta2 glycoprotein I antibodies are associated with some obstetrical complications, mainly preeclampsia-eclampsia. Eur J Obstet Gynecol Reprod Biol 73: 37-42.

4. Ordi Ros J, Falga Tirado C, Monegal Ferran F, Selva O'Callaghan A, Perez Peman P, et al. (1995) [Anti-beta 2 glycoprotein I antibodies. Relationship with antiphospholipid antibodies and thrombosis]. Med Clin (Barc) 104: 245-248.

5. Alijotas-Reig J, Ferrer-Oliveras R, Rodrigo-Anoro MJ, Farran-Codina I, Cabero-Roura L, et al. (2010) Anti-beta(2)-glycoprotein-I and anti-phosphatidylserine antibodies in women with spontaneous pregnancy loss. Fertil Steril 93: 2330-2336.

6. Mankai A, Achour A, Thabet Y, Manoubia W, Sakly W, et al. (2012) Anti-cardiolipin and anti-beta 2-glycoprotein I antibodies in celiac disease. Pathol Biol (Paris) 60: 291-295.

7. Roye-Green K, Frederick J, Wharfe G, Choo-Kang E, DaCosta V, et al. (2011) Antiphospholipid and other autoantibodies in a cohort of habitual aborters and healthy multiparous women in Jamaica. Hum Antibodies 20: 1-5.

8. Holc I, Hojs R, Cikes N, Ambrozic A, Cucnik S, et al. (2011) Antiphospholipid antibodies and atherosclerosis: insights from rheumatoid arthritis--a five-year follow-up study. Immunobiology 216: 1331-1337.

9. Filippidou N, Krashias G, Pericleous C, Rahman A, Ioannou Y, et al. (2016) The association between IgG and IgM antibodies against cardiolipin, beta2-glycoprotein I and Domain I of beta2-glycoprotein I with disease profile in patients with multiple sclerosis. Mol Immunol 75: 161-167.

10. Helgadottir LB, Skjeldestad FE, Jacobsen AF, Sandset PM, Jacobsen EM (2012) The association of antiphospholipid antibodies with intrauterine fetal death: a case-control study. Thromb Res 130: 32-37.

11. Hiltunen LM, Laivuori H, Rautanen A, Kaaja R, Kere J, et al. (2009) Blood group AB and factor V Leiden as risk factors for pre-eclampsia: a population-based nested case-control study. Thromb Res 124: 167-173.

12. Zhou YS, Ying ZT, Li R, Zhu J, Li ZG (2011) [Clinical and immunological relevance of antiphospholipid antibodies in patients with lymphoma]. Zhonghua Yi Xue Za Zhi 91: 2607-2610.

13. Tebo AE, Jaskowski TD, Hill HR, Branch DW (2008) Clinical relevance of multiple antibody specificity testing in anti-phospholipid syndrome and recurrent pregnancy loss. Clin Exp Immunol 154: 332-338.

14. Mezzesimi A, Florio P, Reis FM, D'Aniello G, Sabatini L, et al. (2007) The detection of anti-beta2-glycoprotein I antibodies is associated with increased risk of pregnancy loss in women with threatened abortion in the first trimester. Eur J Obstet Gynecol Reprod Biol 133: 164-168.

15. Hasegawa EM, Caleiro MT, Fuller R, Carvalho JF (2009) The frequency of anti-beta2-glycoprotein I antibodies is low and these antibodies are associated with pulmonary hypertension in mixed connective tissue disease. Lupus 18: 618-621.

16. Markic J, Mestrovic M, Valic I, Sapunar A, Bosnjak N (2007) Frequency of anticardiolipin, antinuclear and anti-beta2 glycoprotein I antibodies in children with epilepsy. Coll Antropol 31: 739-742.

17. Adams MJ, Palatinus AA, Harvey AM, Khalafallah AA (2011) Impaired control of the tissue factor pathway of blood coagulation in systemic lupus erythematosus. Lupus 20: 1474-1483.

18. Pasoto SG, Chakkour HP, Natalino RR, Viana VS, Bueno C, et al. (2012) Lupus anticoagulant: a marker for stroke and venous thrombosis in primary Sjogren's syndrome. Clin Rheumatol 31: 1331-1338.

19. Chilcott IT, Margara R, Cohen H, Rai R, Skull J, et al. (2000) Pregnancy outcome is not affected by antiphospholipid antibody status in women referred for in vitro fertilization. Fertil Steril 73: 526-530.

20. Ferrer-Oliveras R, Llurba E, Cabero-Roura L, Alijotas-Reig J (2012) Prevalence and clinical usefulness of antiphospholipid and anticofactor antibodies in different Spanish preeclampsia subsets. Lupus 21: 257-263.

21. Spinillo A, Beneventi F, Ramoni V, Caporali R, Locatelli E, et al. (2012) Prevalence and significance of previously undiagnosed rheumatic diseases in pregnancy. Ann Rheum Dis 71: 918-923.

22. Bruce IN, Clark-Soloninka CA, Spitzer KA, Gladman DD, Urowitz MB, et al. (2000) Prevalence of antibodies to beta2-glycoprotein I in systemic lupus erythematosus and their association with antiphospholipid antibody syndrome criteria: a single center study and literature review. J Rheumatol 27: 2833-2837.

23. Palomo IG, Mujica VE, Alarcon ML, Pereira JG, Vasquez MR (2005) Prevalence of antiphospholipid antibodies is not different in Chilean diabetic patients and normal individuals. J Diabetes Complications 19: 133-137.

24. Tomsic M, Kveder T, Bozic B, Sodin Semrl S, Cucnik S, et al. (2015) Prevalence, significance and predictive value of antiphospholipid antibodies in Crohn's disease. J Immunol Res 21: 6952-6964.

25. Pilarska E, Lemka M, Bakowska A (2010) Thrombomodulin and antibeta2-glycoprotein I in stroke in children. Med Sci Monit 16: Cr348-351.

26. Lenzi M, Bellentani S, Saccoccio G, Muratori P, Masutti F, et al. (1999) Prevalence of non-organ-specific autoantibodies and chronic liver disease in the general population: a nested case-control study of the Dionysos cohort. Gut 45: 435-441.

27. Chakravarty KK, Gray RE, Webley M, Byron MA, Wozniak J (1991) Prevalence of anticardiolipin antibodies in the elderly British population. Postgrad Med J 67: 358-361.

28. Zielinska J, Ryglewicz D, Wierzchowska E, Lechowicz W, Hier DB, et al. (1999) Anticardiolipin antibodies are an independent risk factor for ischemic stroke. Neurol Res 21: 653-657.

29. Ahmed E, Stegmayr B, Trifunovic J, Weinehall L, Hallmans G, et al. (2000) Anticardiolipin antibodies are not an independent risk factor for stroke: an incident case-referent study nested within the MONICA and Vasterbotten cohort project. Stroke 31: 1289-1293.

30. de Godoy JM, Batigalia F, de Godoy MR, Brandao AC, Souza DR (2004) Anticardiolipin antibodies as a risk factor of atherosclerosis in intermittent claudication. Angiology 55: 357-359.

31. Elefsiniotis IS, Diamantis ID, Dourakis SP, Kafiri G, Pantazis K, et al. (2003) Anticardiolipin antibodies in chronic hepatitis B and chronic hepatitis D infection, and hepatitis B-related hepatocellular carcinoma. Relationship with portal vein thrombosis. Eur J Gastroenterol Hepatol 15: 721-726.

32. Cohen SB, Goldenberg M, Rabinovici J, Lidor AL, Dulitzky M, et al. (2000) Anti-cardiolipin antibodies in fetal blood and amniotic fluid derived from patients with the anti-phospholipid syndrome. Hum Reprod 15: 1170-1172.

33. Zivkovic M, Zlatanovic M, Zlatanovic G, Djordjevic-Jocic J, Cekic S (2011) Anticardiolipin antibodies in patients with Behcet's disease. Bosn J Basic Med Sci 11: 58-61.

34. Sthoeger ZM, Fogel M, Smirov A, Ergas D, Lurie Y, et al. (2000) Anticardiolipin autoantibodies in serum samples and cryoglobulins of patients with chronic hepatitis C infection. Ann Rheum Dis 59: 483-486.

35. Rollino C, Boero R, Elia F, Montaruli B, Massara C, et al. (2004) Antiphospholipid antibodies and hypertension. Lupus 13: 769-772.

36. Bocciolone L, Meroni P, Parazzini F, Tincani A, Radici E, et al. (1994) Antiphospholipid antibodies and risk of intrauterine late fetal death. Acta Obstet Gynecol Scand 73: 389-392.

37. Cervera R, Garcia-Carrasco M, Font J, Ramos M, Reverter JC, et al. (1997) Antiphospholipid antibodies in primary Sjogren's syndrome: prevalence and clinical significance in a series of 80 patients. Clin Exp Rheumatol 15: 361-365.

38. Singer HS, Krumholz A, Giuliano J, Kiessling LS (1997) Antiphospholipid antibodies: an epiphenomenon in Tourette syndrome. Mov Disord 12: 738-742.

39. Pereira de Godoy MR, Cacao JC, Pereira de Godoy JM, Brandao AC, Silva Rossi Souza D (2005) Chagas disease and anticardiolipin antibodies in older adults. Arch Gerontol Geriatr 41: 235-238.

40. Knapik-Kordecka M, Wysokinski WE (2000) Clinical spectrum of Raynaud's phenomenon in patients referred to vascular clinic. Cardiovasc Surg 8: 457-462.

41. Balasa VV, Gruppo RA, Glueck CJ, Wang P, Roy DR, et al. (2004) Legg-Calve-Perthes disease and thrombophilia. J Bone Joint Surg Am 86-a: 2642-2647.

42. Salazar-Paramo M, Jara LJ, Ramos A, Barile L, Machado G, et al. (2002) Longitudinal study of antinuclear and anticardiolipin antibodies in pregnant women with systemic lupus erythematosus and antiphospholipid syndrome. Rheumatol Int 22: 142-147.

43. Donohoe S, Geary M, Kingdom JC, Jauniaux E, Purdy G, et al. (1999) Maternal cardiolipin, beta 2-glycoprotein-I and prothrombin antibody expression in high-risk pregnancies with bilateral abnormal uterine artery Doppler waveforms. Ultrasound Obstet Gynecol 13: 317-322.

44. Oksuzoglu G, Bayraktar Y, Arslan S, Celik I, Arslan M, et al. (2003) Portal vein thrombosis in cirrhotics: related with anticardiolipin antibodies? Hepatogastroenterology 50: 1527-1530.

45. Koren-Michowitz M, Eting E, Rahimi-Levene N, Garach-Jehoshua O, Volcheck Y, et al. (2005) Protein Z levels and central retinal vein or artery occlusion. Eur J Haematol 75: 401-405.

46. Altintas O, Yuksel N, Sonmez GT, Ozkan B, Altintas L, et al. (2012) Serum antiphospholipid antibody levels in pseudoexfoliation. J Glaucoma 21: 326-330.

47. Aggarwal R, Ravishankar B, Misra R, Aggarwal A, Dwivedi S, et al. (1998) Significance of elevated IgG anticardiolipin antibody levels in patients with Budd-Chiari syndrome. Am J Gastroenterol 93: 954-957.

48. Tan L, Zhang Y, Peng W, Chen J, Li H, et al. (2014) Detection of anti-lactoferrin antibodies and anti-myeloperoxidase antibodies in autoimmune hepatitis: a retrospective study. J Immunoassay Immunochem 35: 388-397.

49. Nie H, Wang YY, Wang Y, Shi J, Chen WX (2012) [Correlative analysis of different HBV genotypes and autoantibodies in hepatitis B patients]. Zhonghua Gan Zang Bing Za Zhi 20: 448-452.

50. Iijima T, Tada H, Hidaka Y, Mitsuda N, Murata Y, et al. (1997) Effects of autoantibodies on the course of pregnancy and fetal growth. Obstet Gynecol 90: 364-369.

51. Melguizo Madrid E, Gonzalez-Rodriguez C, Avila-Garcia MG, Arrobas-Velilla T, Fernandez-Riejos P (2017) Zenit RA evaluation, a solid-phase chemiluminescence immunoassay for detection of anti-cellular antibodies. Bioanalysis 9: 435-445.

52. Bentow C, Swart A, Wu J, Seaman A, Manfredi M, et al. (2013) Clinical performance evaluation of a novel rapid response chemiluminescent immunoassay for the detection of autoantibodies to extractable nuclear antigens. Clin Chim Acta 424: 141-147.

53. Deming FP, Al-Hashimi I, Haghighat N, Hallmon WW, Kerns DG, et al. (2007) Comparison of salivary calmodulin binding proteins in Sjogren's syndrome and healthy individuals. J Oral Pathol Med 36: 132-135.

54. Shovman O, Gilburd B, Barzilai O, Shinar E, Larida B, et al. (2005) Evaluation of the BioPlex 2200 ANA screen: analysis of 510 healthy subjects: incidence of natural/predictive autoantibodies. Ann N Y Acad Sci 1050: 380-388.

55. Qi C, Wang L, Zhang M, Shao X, Chang X, et al. (2015) Serum Renalase Levels Correlate with Disease Activity in Lupus Nephritis. PLoS One 10: e0139627.

56. Liang P, Tang Y, Fu S, Lv J, Liu B, et al. (2015) Basophil count, a marker for disease activity in systemic lupus erythematosus. Clin Rheumatol 34: 891-896.

57. Zhao L, Jiang Z, Jiang Y, Ma N, Wang K, et al. (2013) IL-22+CD4+ T-cells in patients with active systemic lupus erythematosus. Exp Biol Med (Maywood) 238: 193-199.

58. Caglar E, Ugurlu S, Ozenoglu A, Can G, Kadioglu P, et al. (2009) Autoantibody frequency in celiac disease. Clinics (Sao Paulo) 64: 1195-1200.

59. Kutukculer N, Yuksel SE, Aksu G, Alper S (2005) Autoantibodies other than antineutrophil cytoplasmic antibodies are not positive in patients with psoriasis vulgaris. J Dermatol 32: 179-185.

60. Giardina E, Triolo G, Accardo-Palumbo A, Carbone MC, Gancitano V, et al. (1997) Anti-single-stranded DNA antibody in the sera of patients with type 2 diabetes mellitus. Relation to vascular complications. Acta Diabetol 34: 39-41.

61. Buskila D, Berezin M, Gur H, Lin HC, Alosachie I, et al. (1995) Autoantibody profile in the sera of women with hyperprolactinemia. J Autoimmun 8: 415-424.

62. Piura B, Tauber E, Dror Y, Sarov B, Buskila D, et al. (1991) Antinuclear autoantibodies in healthy nonpregnant and pregnant women and their offspring. Am J Reprod Immunol 26: 28-31.

63. Sarvas H, Gripenberg M, Leirisalo-Repo M (1985) Anti-DNA antibodies: the choice of assays for routine diagnostic work. Acta Pathol Microbiol Immunol Scand C 93: 13-18.

64. Schott K, Uhl A, Batra A, Bartels M, Eusterschulte B, et al. (1996) Antinuclear antibodies in schizophrenia and major depressive disorder - a lasting puzzle. Eur Psychiatry 11: 263-267.

65. Pradhan VD, Patwardhan MM, Ghosh K (2010) Anti-nucleosome antibodies as a disease marker in systemic lupus erythematosus and its correlation with disease activity and other autoantibodies. Indian J Dermatol Venereol Leprol 76: 145-149.

66. Chretien P, Monier JC, Oksman F, San Marco M, Escande A, et al. (2003) Autoantibodies and human immunodeficiency viruses infection: a case-control study. Clin Exp Rheumatol 21: 210-212.

67. Chen CS, Shi GY (1994) [Detection of serum anti-histone antibodies in patients with ankylosing spondylitis and its clinical significance]. Zhonghua Nei Ke Za Zhi 33: 382-384.

68. Zandman-Goddard G, Gilburd B, Shovman O, Blank M, Berdichevski S, et al. (2005) The homogeneous multiplexed system--a new method for autoantibody profile in systemic lupus erythematosus. Clin Dev Immunol 12: 107-111.

69. Dharamsi JW, Victor S, Aguwa N, Ahn C, Arnett F, et al. (2013) Morphea in adults and children cohort III: nested case-control study--the clinical significance of autoantibodies in morphea. JAMA Dermatol 149: 1159-1165.

70. Putova I, Dostal C, Becvar R (2007) Prevalence of antinucleosome antibodies by enzyme-linked immunosorbent assays in patients with systemic lupus erythematosus and other autoimmune systemic diseases. Ann N Y Acad Sci 1109: 275-286.

71. Samsami DA, Razmjoei P, Parsanezhad ME (2014) Serum Levels of Anti-histone and Anti-double-Strand DNA Antibodies Before and After Laparoscopic Ovarian Drilling in Women with Polycystic Ovarian Syndrome. J Obstet Gynaecol India 64: 47-52.

72. Wu O, Liu HH, Li WX, Zhang N, Wang Q, et al. (2008) Serum soluble nucleosome and the broad family of antinucleosome antibodies are associated with organ and tissue damage in systemic lupus erythematosus in a Chinese population. Clin Exp Dermatol 33: 160-163.

73. Pradhan VD, Badakere SS, Ghosh K, Pawar AR (2004) Spectrum of anti-neutrophil cytoplasmic antibodies in patients with pulmonary tuberculosis overlaps with that of Wegener's granulomatosis. Indian J Med Sci 58: 283-288.

74. Op De Beeck K, Vermeersch P, Verschueren P, Westhovens R, Marien G, et al. (2012) Antinuclear antibody detection by automated multiplex immunoassay in untreated patients at the time of diagnosis. Autoimmun Rev 12: 137-143.

75. Eriksson C, Kokkonen H, Johansson M, Hallmans G, Wadell G, et al. (2011) Autoantibodies predate the onset of systemic lupus erythematosus in northern Sweden. Arthritis Res Ther 13: R30.

76. Gilburd B, Abu-Shakra M, Shoenfeld Y, Giordano A, Bocci EB, et al. (2004) Autoantibodies profile in the sera of patients with Sjogren's syndrome: the ANA evaluation--a homogeneous, multiplexed system. Clin Dev Immunol 11: 53-56.

77. Shen CY, Hsieh SC, Yu CL, Wang JY, Lee LN, et al. (2013) Autoantibody prevalence in active tuberculosis: reactive or pathognomonic? BMJ Open 3.

78. Shin HD, Park BL, Kim LH, Lee HS, Kim TY, et al. (2004) Common DNase I polymorphism associated with autoantibody production among systemic lupus erythematosus patients. Hum Mol Genet 13: 2343-2350.

79. Tozkir H, Pamuk ON, Duymaz J, Gurkan H, Yazar M, et al. (2016) Increased frequency of class I and II anti-human leukocyte antigen antibodies in systemic lupus erythematosus and scleroderma and associated factors: a comparative study. Int J Rheum Dis 19: 1304-1309.

80. Zhen X, Qiao J, Li R, Wang L, Liu P (2014) Serologic autoimmunologic parameters in women with primary ovarian insufficiency. BMC Immunol 15: 11.

81. Etemadifar M, Fatemi A, Hashemijazi H, Kazemizadeh A (2013) Is it necessary to perform connective tissue disorders laboratory tests when a patient experiences the first demyelinating attack? J Res Med Sci 18: 617-620.

82. Wikman A, Fagergren A, Gunnar OJS, Lundahl J, Jacobson SH (2003) Monocyte activation and relationship to anti-proteinase 3 in acute vasculitis. Nephrol Dial Transplant 18: 1792-1799.

83. Flores-Suarez LF, Cabiedes J, Villa AR, van der Woude FJ, Alcocer-Varela J (2003) Prevalence of antineutrophil cytoplasmic autoantibodies in patients with tuberculosis. Rheumatology (Oxford) 42: 223-229.

84. Wu YY, Hsu TC, Chen TY, Liu TC, Liu GY, et al. (2002) Proteinase 3 and dihydrolipoamide dehydrogenase (E3) are major autoantigens in hepatitis C virus (HCV) infection. Clin Exp Immunol 128: 347-352.

85. Kuryliszyn-Moskal A, Klimiuk PA, Sierakowski S (2001) Serum autoantibodies profile and increased levels of circulating intercellular adhesion molecule-1: a reflection of the immunologically mediated systemic vasculopathy in rheumatic diseases? Arch Immunol Ther Exp (Warsz) 49: 423-430.

86. Kovacs L, Szabo J, Molnar K, Kovacs A, Pokorny G (1999) Antineutrophil cytoplasmic antibodies and other immunologic abnormalities in patients with habitual abortion. Am J Reprod Immunol 41: 264-270.
